# Scientific American

**Published:** 1874-02

**Authors:** 


Scientific American.—This truly scientific and highly practical weekly seems to gather strength year by year. Its pages are always well filled with scientific and useful reading as well as being finely illustrated. We can recommend it to our readers as a journal they will appreciate and prize. Price $3.00. Munn & Co., 37 Park Row, New York.

